# Supplementary material for: Content-rich biological network constructed by mining PubMed abstracts
Source: BMC Bioinformatics. 2004 Oct 8;5:147. doi: 10.1186/1471-2105-5-147 (PMC528731; doi:10.1186/1471-2105-5-147)
Supplement: Additional File 2 — The original results of the above study (non-essential files are deleted to keep the file size under the limit set by BMC bioinformatics). [file 1471-2105-5-147-S2.bz2 › chilibotAdditionalFile2/dip05/211ID7768894E717/html/CSNK2A1_CSNK2B.html]

 


 **CSNK2A1** and **CSNK2B** 
  
Found 143 abstracts in PubMed, retrieved 05.  
 

 What does Google say? 
 PDF only 
| .edu only 

---

**Interactive relationship** (e.g. stimulation, inhibition, etc)

**Non-interactive relationship** (e.g. studied together, co-existance, homology, etc.)

- The recombinant N was phosphorylated by BHK cellular extracts and by purified CK II  [ **CSNK2B** ]   [ **CSNK2A1** ] .  Ref: 12711319 Biochem Biophys Res Commun, 2003
- Thus, our data demonstrate that cellular CK II  [ **CSNK2B** ]   [ **CSNK2A1** ]  phosphorylates rabies virus N.  Ref: 12711319 Biochem Biophys Res Commun, 2003
- In addition, the phosphorylation of the recombinant N in vitro can be blocked by a CK II  [ **CSNK2B** ]   [ **CSNK2A1** ]  inhibitor, heparin.  Ref: 12711319 Biochem Biophys Res Commun, 2003
- The reading frame encodes a polypeptide consisting of 526 amino acid residues, containing a number of DNA binding motifs and phosphorylation sites for PKC, CK II  [ **CSNK2B** ]   [ **CSNK2A1** ] , and p34cdc2.  Ref: 12736779 J Mol Med< MedlineTA>J Mol Med, 2003
- Characterization of a CK II  [ **CSNK2B** ]   [ **CSNK2A1** ]  protein kinase from etiolated oat seedlings.  Ref: 12243354 Mol Cells, 2002
- hondai on the activities of four distinct Ser Thr protein kinases two casein kinases CK I and CK II  [ **CSNK2B** ]   [ **CSNK2A1** ] , A kinase and C kinase were determined in vitro.  Ref: 12612451 Biol Pharm Bull, 2003
- These results, therefore, indicate that the purified kinase is a CK II  [ **CSNK2B** ]   [ **CSNK2A1** ]  protein kinase and may be involved in the regulation of DNA topoisomerase I activity.  Ref: 12243354 Mol Cells, 2002
- Furthermore, N phosphorylation in the virus infected cells can be inhibited by a CK II  [ **CSNK2B** ]   [ **CSNK2A1** ]  specific inhibitor, dichloro beta D ribofuranosyl benzimidazole.  Ref: 12711319 Biochem Biophys Res Commun, 2003
- The monoclonal antibodies CK II  [ **CSNK2B** ]   [ **CSNK2A1** ]  and A45 B B3 were used with the immuno cytochemical standard method for detecting IDT BM.  Ref: 12678519 Int J Surg Investig, 2000
